# Seroreactivity against lytic, latent and possible cross-reactive EBV antigens appears on average 10 years before MS induced preclinical neuroaxonal damage

**DOI:** 10.1136/jnnp-2023-331868

**Published:** 2023-10-06

**Authors:** Daniel Jons, Viktor Grut, Tomas Bergström, Henrik Zetterberg, Martin Biström, Martin Gunnarsson, Magnus Vrethem, Nicole Brenner, Julia Butt, Kaj Blennow, Staffan Nilsson, Ingrid Kockum, Tomas Olsson, Tim Waterboer, Peter Sundström, Oluf Andersen

**Affiliations:** 1 Department of Clinical Neuroscience, Institute of Neuroscience and Physiology, The Sahlgrenska Academy, University of Gothenburg, Göteborg, Sweden; 2 Department of Clinical Science, Neurosciences, Umeå University, Umeå, Sweden; 3 Department of Infectious Diseases, Institute of Biomedicine, the Sahlgrenska Academy, University of Gothenburg, Göteborg, Sweden; 4 Department of Clinical Microbiology, Sahlgrenska University Hospital, Göteborg, Sweden; 5 Department of Psychiatry and Neurochemistry, Institute of Neuroscience and Physiology, the Sahlgrenska Academy, University of Gothenburg, Göteborg, Sweden; 6 Clinical Neurochemistry Laboratory, Sahlgrenska University Hospital, Mölndal, Sweden; 7 Department of Neurology, Faculty of Medicine and Health, Örebro University, Örebro, Sweden; 8 Department of Neurology and Department of Biomedical and Clinical Sciences, Linköping University, Linköping, Sweden; 9 Infections and Cancer Epidemiology, Infection, Inflammation and Cancer Research Program, German Cancer Research Center, Heidelberg, Germany; 10 Mathematical Sciences, Chalmers University of Technology, Göteborg, Sweden; 11 Department of Laboratory Medicine, Institute of Biomedicine, Sahlgrenska Academy, University of Gothenburg, Goteborg, Sweden; 12 Department of Clinical Neuroscience, The Karolinska Neuroimmunology & Multiple Sclerosis Center, Center for Molecular Medicine, Karolinska Institute, Stockholm, Sweden

**Keywords:** MULTIPLE SCLEROSIS, VIROLOGY

## Abstract

**Background:**

Multiple sclerosis (MS) and presymptomatic axonal injury appear to develop only after an Epstein-Barr virus (EBV) infection. This association remains to be confirmed across a broad preclinical time range, for lytic and latent EBV seroreactivity, and for potential cross-reacting antigens.

**Methods:**

We performed a case–control study with 669 individual serum samples obtained before clinical MS onset, identified through cross-linkage with the Swedish MS register. We assayed antibodies against EBV nuclear antigen 1 (EBNA1), viral capsid antigen p18, glycoprotein 350 (gp350), the potential cross-reacting protein anoctamin 2 (ANO2) and the level of sNfL, a marker of axonal injury.

**Results:**

EBNA1 (latency) seroreactivity increased in the pre-MS group, at 15–20 years before clinical MS onset, followed by gp350 (lytic) seroreactivity (p=0.001–0.009), ANO2 seropositivity appeared shortly after EBNA1-seropositivity in 16.7% of pre-MS cases and 10.0% of controls (p=0.001).With an average lag of almost a decade after EBV, sNfL gradually increased, mainly in the increasing subgroup of seropositive pre-MS cases (p=8.10^−5^ compared with non-MS controls). Seropositive pre-MS cases reached higher sNfL levels than seronegative pre-MS (p=0.038). In the EBNA1-seropositive pre-MS group, ANO2 seropositive cases had 26% higher sNfL level (p=0.0026).

**Conclusions:**

Seroreactivity against latent and lytic EBV antigens, and in a subset ANO2, was detectable on average a decade before the appearance of a gradually increasing axonal injury occurring in the last decade before the onset of clinical MS. These findings strengthen the hypothesis of latent EBV involvement in the pathogenesis of MS.

**WHAT IS ALREADY KNOWN ON THIS TOPIC**

- Five to ten years before the clinical onset of multiple sclerosis (MS), the average serum level of neurofilament light starts to increase, indicating preclinical axonal injuries. According to longitudinal data, reactivity against Epstein-Barr nuclear antigen 1 (EBNA1) increases before axonal injury that occurs during the last decade before onset, compatible with a role for Epstein-Barr virus (EBV) as a trigger for MS. The search for a possible autoantigen in MS has been unrewarding, although recent data support the chloride channel protein anoctamin 2 (ANO2) as an autoantigen for a subgroup of patients with MS.

**WHAT THIS STUDY ADDS**

- There is an average lag of almost 10 years between the rise in EBV serology and the onset of preclinical axonal injury in MS. Neutralising antibodies against the EBV glycoprotein gp350 indicates that the increased immunoreactivity against EBV depends on a preceding primary Epstein-Barr infection. Antibodies against ANO2 appear in a subset soon after the Epstein-Barr infection, associated with increasing risk of preclinical MS-induced axonal injury.

**HOW THIS STUDY MIGHT AFFECT RESEARCH, PRACTICE OR POLICY**

- The results provide a template for research on other, for example, radiological, immunological and therapeutic aspects of preclinical MS. Vaccination trials against EBV are ongoing and the present results are relevant for follow-up regarding preclinical MS and MS incidence after these trials.

## Introduction

Several pieces of evidence support the involvement of Epstein-Barr virus (EBV) in multiple sclerosis (MS) pathogenesis. Among them is that the risk of MS increases after infectious mononucleosis (IM).^1^ In addition, serological studies suggest that a primary EBV infection is a prerequisite for MS,^2 3^ and an increased EBV seroresponse, apparently mainly against EBV nuclear antigen 1 (EBNA1),^4–7^ is detectable several years before clinical onset of MS.^7 8^ Patients with MS may have experienced a severe and possibly atypical EBV primary infection.^7 9^ Thus, serological analyses of archived serum specimens from individuals who later developed MS have revealed an association of increased EBNA1 antibody titres and MS risk from the age of 25 onward.^10^ Investigators in Sweden reported that a primary EBV infection in childhood or adolescence was associated with relative protection against MS, whereas a later primary infection was associated with increased MS risk.^11^ In a pivotal US study,^12^ 766 of 801 (96%) of pre-MS patients were EBV seropositive in a baseline sample obtained at a median 8 (range 3–17) years before clinical MS onset. All but one of the 35 remaining EBV-seronegative individuals became EBV seropositive before onset. Moreover, in 30 cases and 30 controls randomly selected from the same cohort, elevation of serum neurofilament light (sNfL) protein, a marker of axonal damage and the first objective sign of MS-induced injury, was observed at a median 6 (range 4–10) years before clinical MS onset.^13^ Similarly, in our previous report on sNfL in successive 5-year periods,^14^ average sNfL levels showed an increasing trend starting in specimens obtained 5–10 years before clinical MS onset. Combining their consecutive EBV and sNfL data the US investigators showed that EBV seroconversion events preceded incipient elevation of sNfL, implicating EBV infection as the leading cause of MS.^12^ The age distribution in these studies reflected that of active military personnel, and the crucial observation of sNfL elevation following EBV seroconversion depended on the absence of sNfL elevation in the random sample of the original cohort. The association between EBV conversion and sNfL elevation remains to be confirmed in a broader population with a wider age distribution.

Plasma antibody assays and absorption tests have uncovered an EBNA1 epitope that cross-reacts with the central nervous system protein anoctamin 2 (ANO2) in 14.6% of patients with MS and 7.8% of controls.^15 16^ Reactivity to a similar EBNA1 peptide was reported as part of polyspecific autoimmunity during acute IM.^17 18^

Here, we assessed whether the appearance of EBV seroreactivity in the presymptomatic phase of MS precedes axonal injury detectable with sNfL and whether it is associated with incipient autoreactivity against one reported possible MS autoantigen (ANO2). Leveraging a large repository of material from Swedish university hospital biobanks, we explored seroreactivity against a latency EBV antigen (EBNA1) and two lytic antigens, viral capsid antigen p18 (VCAp18) and glycoprotein 350 (gp350), the latter of which is the major neutralising antibody. We compare the preclinical distribution of sNfL elevation between pre-MS groups with negative and positive EBV and ANO2 seroreactivity.

## Material and methods

### Participants

This nested case–control study used presymptomatically collected blood samples from 669 individuals (pre-MS group) who later received a diagnosis of relapsing-remitting MS and 669 matched controls, as described previously.^11 19^ These individuals were identified from the Swedish MS register,^20^ and a local MS database in Umeå, Sweden. The MS register contained 11 146 patients (www.neuroreg.se), and the Umeå database contained 2887 patients. The register data were crosslinked with serum samples stored in six Swedish biobanks, containing aliquots remaining after clinical microbiological analyses performed at the university hospitals of Skåne, Gothenburg, Örebro, Linköping and Umeå, and at the Public Health Agency of Sweden in Stockholm. Individuals with relapsing-remitting MS whose presymptomatic blood sample was obtained when they were younger than 40 years were included. Controls were matched 1:1 for biobank, sex, date of blood sampling and date of birth, in decreasing priority. Controls were matched with a mean absolute difference of 6 days for date of sampling and 152 days for age at sampling.^11^ Because of the scarcity of sera, sNfL analyses were limited to 519 case–control pairs and gp350 analyses to 570 of the 669 case–control pairs. The median sampling age of the study population was 25 years (IQR 21–29 years; range: 2–39 years), and 84% of the participants were female. Some biobanks included samples collected during early pregnancy, with a higher proportion of female participants. The median time from sampling to clinical MS onset was 8 years (IQR: 4–13 years; see table 1).

**Table 1:** Characteristics of pre-MS cases

Pre-MS samples					
Time from sampling until MS onset	1–33 years	<5 years	5–10 years	10–15 years	15–33 years
N	669	222	188	147	112
Female sex, %	84	87	84	77	86
Median age (IQR) at sampling, years	25 (21–29)	26 (22–31)	25 (20–29)	25 (21–29)	22 (19–26)
Median age (IQR) at MS onset, years	33 (28–40)	29 (25–33)	32 (27–37)	37 (32–42)	43 (38–46)

Samples analysed for sNfL					
Time from sampling until MS onset	1–32 years	<5 years	5–10 years	10–15 years	15–32 years
n	519	112	163	137	107
Female sex, %	82	82	85	77	85
Median age (IQR) at sampling, years	25 (21–29)	27 (23–32)	25 (20–29)	25 (21–29)	22 (19–26)
Median age (IQR) at MS onset, years	35 (29–41)	30 (26–34)	32 (28–37)	37 (32–42)	43 (38–46)

Samples analysed for gp350					
Time from sampling until MS onset	1–33 years	<5 years	5–10 years	10–15 years	15–33 years
N	570	189	159	124	98
Female sex, %	84	88	84	77	84
Median age (IQR) at sampling, years	25 (21–29)	27 (23–31)	25 (20–29)	25 (21–28)	22 (18–26)
Median age (IQR) at MS onset, years	34 (28–40)	29 (25–33)	32 (28–37)	37 (33–42)	43 (38–46)

Controls were matched and had the same characteristics. MS onset means clinical MS onset.

MS, multiple sclerosis; sNfL, serum neurofilament light.

The time of clinical MS onset was extracted from the Swedish national MS registry and Umeå medical records. These registers provide prospective data on several clinical variables, but the recorded onset of MS, defined as the first symptom suggestive of a demyelinating event, was often ascertained retrospectively.

Participants gave informed consent, with an opt-out option, to participate in the study before taking part.

### Laboratory methods

The German Cancer Research Centre in Heidelberg, Germany, performed assays of antibodies against EBV antigens EBNA1 trunc (aa 325–641), VCAp18 (aa 1–175)^18 21^ and ANO2 (aa 79–167), using a bead-based multiplex assay as described previously.^22^ Samples were analysed in multiple batches, and interbatch controls were used to correct for batch-related variability, as previously described.^23^ EBV serostatus was determined using published cut-offs of 1800 median fluorescence intensity (MFI) for EBNA1 trunc and 2526 MFI for VCAp18.^21^ Samples were classified as EBV seronegative when values for both EBNA1 and VCAp18 fell below the published cut-offs. EBV seropositivity was defined as either EBNA1 or VCAp18 above these cut-offs and ANO2 seropositivity as above a cut-off of 420 MFI.^16^

The Department of Clinical Microbiology at Sahlgrenska University Hospital, Gothenburg, Sweden, performed the analyses of gp350 antibodies. These antibodies were analysed using ELISA with a DNA construct encoding the full 860-aa extracellular domain of gp350 as the antigen, as previously described.^24^ Plates were coated with antigen at a concentration of 2 µg/mL, and all samples were diluted 1/200 and analysed in duplicate. Case–control samples were analysed on the same plates.

The Clinical Neurochemistry Laboratory at Sahlgrenska University Hospital, Gothenburg, Sweden, measured sNfL levels using single-molecule array (Simoa) technology and the NF-LIGHT assay on an HD-X Analyzer, according to manufacturer instructions (Quanterix, Billerica, Massachusetts, USA). All samples were measured by board-certified laboratory technicians in a single round of experiments using a single batch of reagents. A quality-control sample with an sNfL concentration of 6.8 pg/mL resulted in 11.2% repeatability and 11.2% intermediate precision. In a quality-control sample with a concentration of 50.3 pg/mL, repeatability was 6.9%, and intermediate precision was 10.6%.

### Statistical methods

Paired t-tests were used to compare sNfL levels between pre-MS samples and controls for EBNA1 seropositive and EBNA1 seronegative samples. To test the distributional difference of sNfL between those with pre-MS who were EBV seropositive and EBV seronegative, we counted the number X of the 504 EBV-seropositive samples with a higher sNfL level than the highest of the 39 EBV seronegative sample. Age adjustment was based on the age dependency in the controls. P values were calculated using combinatorics. Assuming equal distributions this gives P(X≥k)=1/(n+m choose k) and the expected number of X is 504.1/(39+1).

Paired t-tests were used to compare seroreactivity and log sNfL between pre-MS cases and their matched controls for the whole sample and for 5-year time groups until clinical MS onset, and between subgroups with and without EBV seroreactivity. Individual delta values (pre-MS minus matched control) were calculated and plotted against time to MS onset, and relationships were estimated with smooth regression analysis using the locally estimated scatterplot smoothing (ie, loess) regression function in R. Effect size (Cohen’s d) was calculated for each 5-year time group. Pearson’s correlations between each of the three antibodies and log sNfL were analysed for pre-MS cases.

Matched pairs that were discordant for EBV seropositivity or ANO2 seropositivity were compared with one-sided McNemar’s test using the binomial distribution. Age distributions in ANO2-seropositive and ANO2-seronegative samples were compared with the Kolmogorov-Smirnov test. The association between ANO2 seropositivity and the level of sNfL was analysed with age-adjusted analysis of covariance (ANCOVA), stratified for EBNA1 seropositivity. All statistical analyses were performed in R.

## Results

### EBV serostatus

Of the 669 pre-MS case–control pairs, 628 samples in the pre-MS group (94%) and 623 control samples (93%) were EBV seropositive. Approaching MS onset, a higher percentage of the pre-MS cases turned EBV seropositive than the matched controls. Of 222 pre-MS cases sampled in the last 5-year period before clinical onset, 2 were EBV seronegative, compared with 12 in the control group (p=0.01; table 2). Samples acquired earlier than 5 years before MS onset did not differ significantly (table 2).

**Table 2:** Discordant pairs for EBV seropositivity

Years to clinical MS onset	Pre-MS pos control neg	Pre-MS neg control pos	P value*	Both EBV pos	Both EBV neg	N (pairs)
All	38	33	0.64	590	8	669
<5	12	2	0.01	208	0	222
5–10	12	6	0.24	167	3	188
>10	14	25	0.11	215	5	259

Discordant pairs: one of the samples (case or control) was EBV seronegative and the other EBV seropositive, as defined in the Methods section.

*McNemar test.

EBV, Epstein-Barr virus; MS, multiple sclerosis; neg, negative; pos, positive.

### EBV serostatus and sNfL

Among the EBV-seropositive samples, the sNfL levels were higher in the pre-MS group than in the control group (ratio: 1.14; 95% CI 1.07 to 1.22; p=8.10^−5^) for the whole study time frame, and for the last 10-year period before clinical MS onset (ratio:1.18; 95% CI 1.09 to 1.28; p=8.10^−5^). Among the EBV-seronegative samples, which was a much smaller group, no difference was found, with a pre-MS:control sNfL ratio of 1.12 for the whole study timeframe (n=36; 95% CI: 0.93 to 1.34; p=0.22) and 1.02 (n=7; 95% CI: 0.52 to 1.99; p=0.95) for the last 10-year period before MS onset.

**Figure 1:**
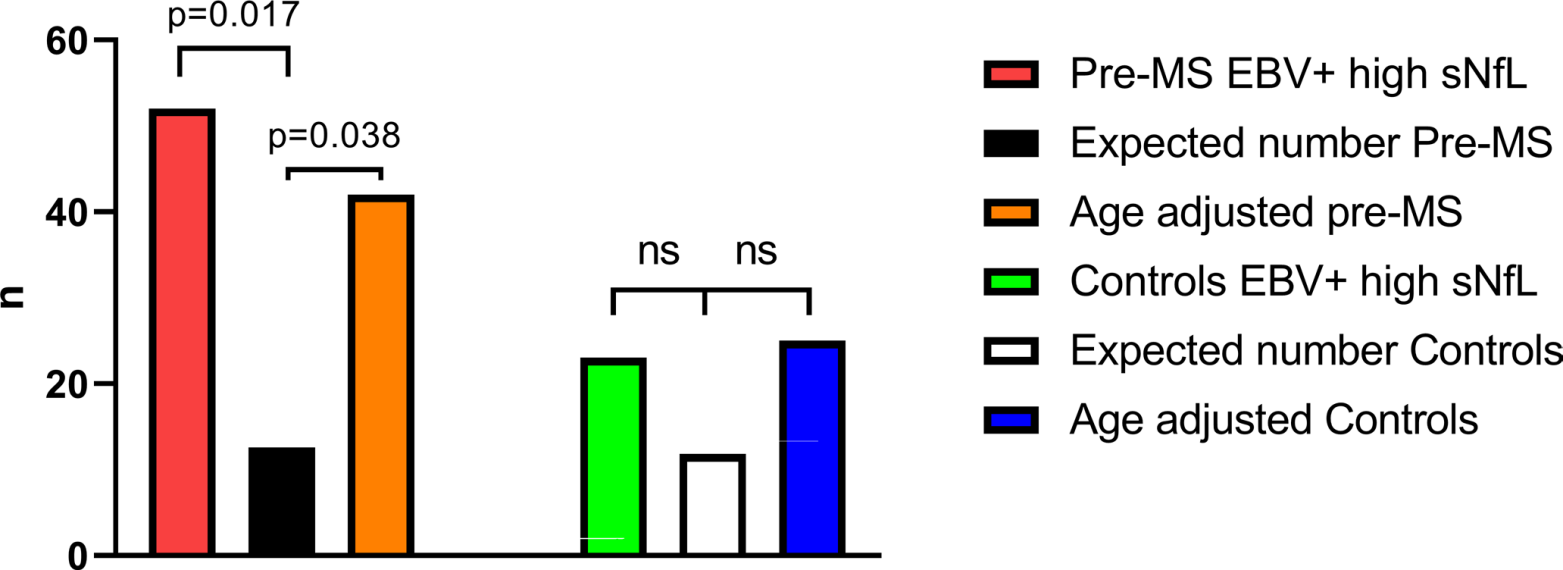
High sNfL values were predominately found in EBV-positive pre-MS samples. Number of samples in the EBV-seropositive pre-MS group with an sNfL concentration higher than the highest value in the EBV seronegative pre-MS group (13.9 pg/mL before age adjustment): n=52 before and n=42 after age adjustment. Compared with the expected value (504/ (39+1)=12.6) under the null hypothesis of equal distribution, the difference for sNfL was significant both before and after age adjustment, indicating a higher percentage of samples with elevated sNfL in the EBV-seropositive versus EBV-seronegative pre-MS group. The same calculation comparing EBV seropositives and negatives in the matched control group showed no significant differences. EBV, Epstein-Barr virus; ns, not significant; pre-MS, before multiple sclerosis diagnosis; sNfL, serum neurofilament light.

Within the pre-MS group, 39 of 543 pre-MS samples with sNfL available were EBV seronegative. The highest sNfL concentration in this subgroup was 13.9 pg/mL. However, 52 of the 504 EBV-seropositive pre-MS cases (10%) had sNfL values above the cut-off of 13.9 pg/mL. That proportion was significantly higher than the expected value calculated from the distribution in the EBV-seronegative group (2.5%=12.6, p=0.017, age-adjusted p=0.038) (figure 1). For controls, the highest sNfL level in the EBV-seronegative group was 14.7 pg/mL. Of the 495 EBV-seropositive controls, 23 (4.6%) had sNfL values>14.7 pg/mL. This value did not differ significantly from the expected value (n=11.8) calculated from the distribution in EBV-seronegative controls (p=0.17, age-adjusted p=0.13).

### EBV antibody levels

Among EBV-seropositive samples, EBNA1, VCAp18 and gp350 seroreactivities were significantly higher in the pre-MS group than in the matched control group (p*=*0.005 to <0.0001; table 3).

**Table 3:** EBV and ANO2 antibodies and sNfL in EBNA1-positive case–control pairs

		Pre-MS		Control persons		
	N (pairs)	Median	IQR	Median	IQR	P value*
EBNA1 (kMFI)	557	9.1	(7.6–11)	8.2	(6.2–10)	<0.0001
VCAp18 (kMFI)	557	7.7	(5.2–9,9)	7.2	(4.8–9.3)	0.005
Gp350 (OD)	477	1.8	(0.9–2.6)	1.4	(0.8–2.3)	0.0001
sNfL (pg/ml)	427	6.6	(4.9–9.4)	6.0	(4.5–8.0)	0.0003
ANO2 (kMFI)	557	9.2	(2.4–62)	7.0	(2.3–39.9)	0.01

Only case–control pairs in which both samples were EBNA1 seropositive are included. The ANO2 comparison includes values below the ANO2 cut-off.

*Paired t-tests of EBV-positive (EBNA1) group, including all age groups.

ANO2, anoctamin 2; EBNA1, Epstein-Barr virus nuclear antigen 1; gp350, EBV glycoprotein 350; kMFI, kilo median fluorescence intensity; MS, multiple sclerosis; OD, optical density; sNfL, serum neurofilament light; VCAp18, viral capsid antigen protein 18.

**Figure 2:**
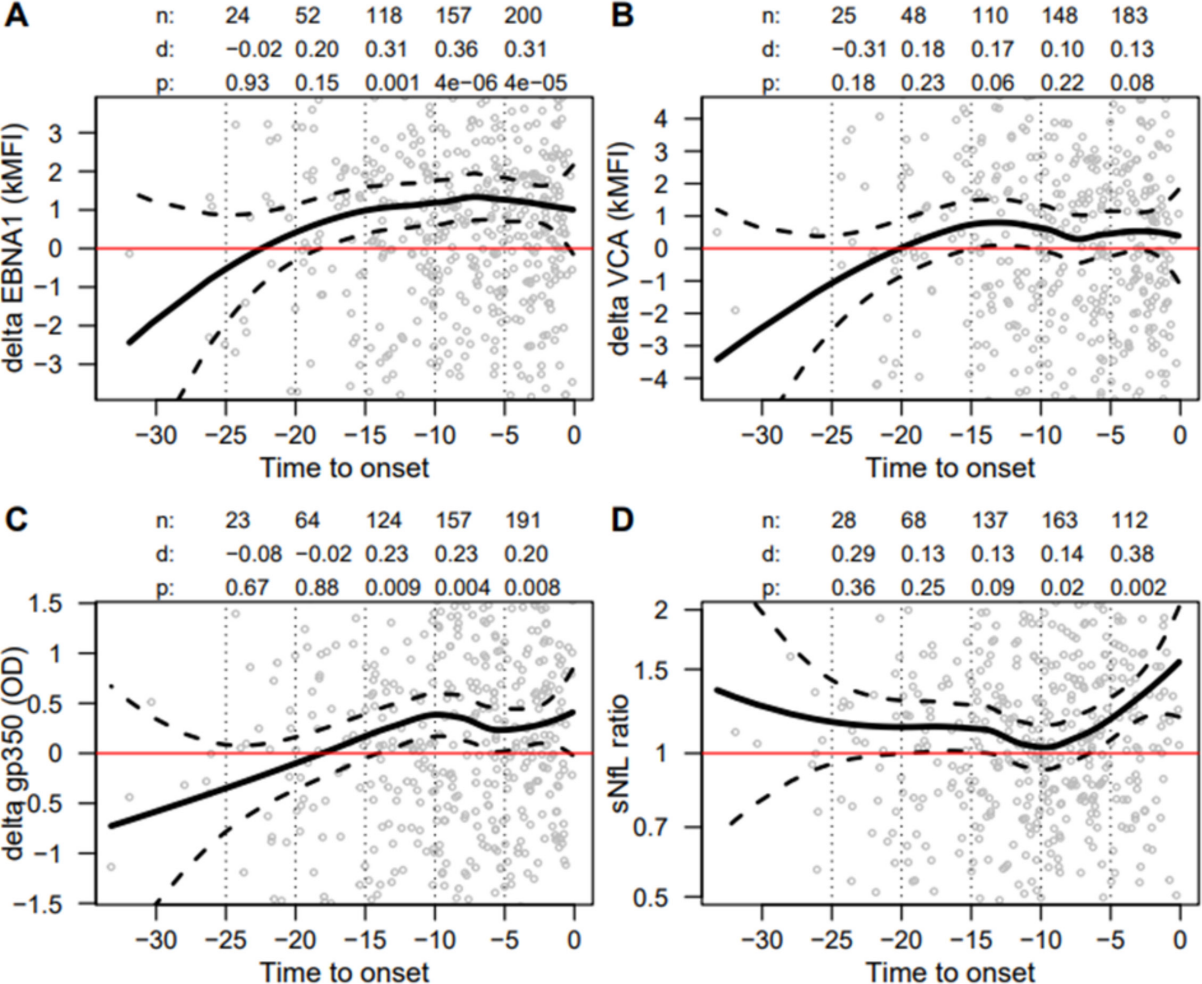
EBV antibodies and sNfL against time to clinical MS onset. Delta values (pre-MS cases–controls) for EBV-positive samples (grey circles) plotted as loess regression (solid black line) against time to clinical MS onset. The y-axis covers±1 SD of scale. Dotted lines show 95% CIs. Numbers above graphs show matched t-tests for 5 years intervals. (A) Pre-MS cases had significantly higher EBNA1 values than controls at 10–15, 5–10 and 0–5 years before clinical MS onset. (B) No significant difference was observed for VCAp18. (C) Pre-MS cases had significantly higher gp350 values than controls at 10–15, 5–10 and 0–5 years before clinical MS onset. (D) Within-pair ratio of sNfL increased in pre-MS cases from approximately 10 years before clinical MS onset and was significant at 0–5 years before clinical MS onset. Definitions: d, effect size (Cohen’s d; ie, mean/SD); EBNA1, Epstein-Barr virus nuclear antigen 1; EBV, Epstein-Barr virus; gp350, glycoprotein 350; loess, locally estimated scatterplot smoothing; kMFI, kilo median fluorescent intensity; MS, multiple sclerosis; pre-MS, before MS diagnosis; n, number of observations in time frame; OD, optical density; sNfL, serum neurofilament light; VCAp18, viral capsid antigen protein 18.

EBNA1 seroreactivity was elevated in pre-MS cases from 15 to 20 years before MS onset and significantly higher than among control samples from 10 to 15 years before clinical MS onset. EBNA1 seroreactivity remained elevated at approximately the same higher level in the intervals of 5–10 and 0–5 years before MS onset (figure 2A). Gp350 seroreactivity displayed a similar pattern, with a possibly later increase. For VCAp18, we detected no significant increase in the corresponding 5-year intervals (figure 2B).

### EBV antibody levels and sNfL levels

In the pre-MS group elevated sNfL was detectable 5–10 years before clinical MS onset, approximately a decade after the increase in EBV seroreactivity (figure 2).

We observed no correlation between the level of sNfL and EBV seroreactivity for EBV-seropositive samples from the complete pre-MS-group (online supplemental figure S1) or in samples acquired 0–5 years before MS onset (data not shown). In EBV-seropositive cases, we observed no correlation between gp350 and EBNA1 seroreactivity (r=−0.001, p=0.98), whereas VCAp18 correlated weakly with EBNA1 and gp350 (online supplemental figure S1).

10.1136/jnnp-2023-331868.supp1 Supplementary data

### ANO2 serostatus in relation to EBNA1 serology

Only one individual in the EBNA1-seronegative group was seropositive for ANO2, with an MFI value immediately above the cut-off. In the EBNA1-seropositive group, ANO2 seropositivity was observed in 16.7% of pre-MS cases and 10.0% of controls (p=0.001).

Focusing on sample pairs collected less than 15 years before clinical MS onset, we studied the role of ANO2 by counting all combinations of positivity for EBNA1 and ANO2 in cases and controls (table 4).

**Table 4:** Concordant and discordant pairs for EBNA1 and ANO2 seropositivity in samples collected <15 years before clinical onset

Matched controls						
		EBNA1−, ANO2-	EBNA1−, ANO2+	EBNA1+, ANO2−	EBNA1+, ANO2+	Total
**Pre-MS**	**EBNA1−, ANO2-**	9	0	**24**	2	35
	**EBNA1−, ANO2+**	0	0	1	0	1
	**EBNA1+, ANO2−**	**39**	0	387	**29**	431
	**EBNA1+, ANO2+**	7	0	**58**	1	91
	**Total**	55	0	449	54	558

Concordant (grey shading) and discordant matched pairs for EBNA1 and ANO2 seropositivity and seronegativity. Showing that in the last 15-year period before clinical MS onset there are more EBNA1 seropositive samples with also seropositive ANO2 in the pre-MS group (n=58) than in the control group (n=29, p=0.002). There are also more EBNA1 seropositive samples in the ANO2 seronegative pre-MS group (n=39) than in ANO2 seronegative control group (n=24, p=0.038). Numbers used in comparisons in bold. P values from McNemar test.

ANO2, anoctamin 2; EBNA1, Epstein-Barr virus nuclear antigen 1; MS, multiple sclerosis.

Of 87 EBNA1-seropositive pairs discordant for ANO2, we found that ANO2 was seropositive in 58 pre-MS cases compared with 29 controls (p=0.002). This finding suggested that ANO2 seropositivity was linked to a further increased risk of subsequent MS in EBNA1-seropositive individuals. Furthermore, of 63 ANO2-seronegative pairs discordant for EBNA1, we found that EBNA1 was seropositive in 39 pre-MS cases compared with 24 controls (p=0.038), suggesting increased risk with EBNA1 seropositivity, independent of ANO2.

The age distribution was almost identical for ANO2-seropositive and ANO2-seronegative samples within EBV-seropositive pre-MS cases and within controls (p=0.68, online supplemental figure S1). If conversion to ANO2 seropositivity had occurred well after EBNA1 seroconversion, a number of individuals would be ANO2 seropositive at a higher age, leading to a right-shifted age distribution. As we identified no difference in the age distribution (online supplemental figure S2), we can infer that ANO2 seropositivity developed shortly after seroconversion to EBV positivity.

For samples positive for both ANO2 and EBNA1, serum ANO2 levels were weakly correlated with EBNA1 (*r*=0.31, p=0.006). This correlation was attenuated and not significant if an outlier was removed (online supplemental figure S3). Among EBNA1-seropositive pre-MS cases, within 15 years from clinical MS onset, ANO2 seropositivity was associated with a 26% higher sNfL (p=0.0026).

## Discussion

Here, we analysed seroreactivity against lytic and latent EBV antigens and against the putative EBNA1-ANO2 cross-antigen, as well as sNfL levels in the presymptomatic phase of MS. We leveraged serum repositories representing a wide age range in a cross-matching procedure with a Swedish national MS register. Our findings show increased EBV antibody levels in seropositive pre-MS individuals from 15 to 20 years before clinical MS onset, followed by increased sNfL from approximately 5–10 years before onset, about a decade after EBV seroreactivity was detected. Antibodies against the putative autoantigen ANO2 appeared almost solely in the EBNA1-seropositive group. This seroreactivity developed soon after EBNA1 seroconversion, but only in a limited proportion of participants: 17% of pre-MS individuals and 10% of matched controls. As expected,^2 3^ the EBV-seronegative subgroup continuously diminished in size during the time leading up to clinical MS onset. Pre-MS individuals with the highest sNfL levels were concentrated in the EBV-seropositive subset.

We relied on one sampling date per individual, without longitudinal information. After an increased EBV antibody level was reached in pre-MS cases 10–15 years before clinical onset, similarly increased levels of antibodies were detected in individuals sampled in the subsequent periods leading up to MS onset. This observation supports the idea that presymptomatically increased EBV antibodies reflect an acute preceding event, conceivably the primary EBV infection, rather than resulting from a proliferating EBV infection, which would be expected to generate increasing EBV seroreactivity along with increasing sNfL levels. A previous study showed an approximately 4-fold higher EBNA1 antibody titre at initial sampling in a small pre-MS group compared with EBV-seropositive controls (table 2 in 25) further supporting that increased EBV seroreactivity before clinical MS onset originates from a primary infection. How many of these primary infections manifested as IM is not known. EBNA1 is a latency antigen, described as a dominant antigen in serological studies in clinical^26^ and presymptomatic MS.^18 27^ VCA antibody reactivity, however, has been associated with other diseases, such as systemic lupus erythematosus,^28^ post-transplantation lymphoproliferative disorders^29^ and Hodgkin’s lymphoma.^30^

We also evaluated gp350 as a major pre-MS lytic antigen with an established neutralising capacity that elicits persistent immunoreactivity in convalescence 10 years after IM compared with asymptomatic primary infection.^31^ We found that neutralising gp350 antibodies did not correlate with antibody levels against latent EBNA1. This lack of correlation between lytic gp350 and latent EBNA1 seroreactivity also has been reported in patients diagnosed with MS.^6^ In the absence of clinically discernible EBV reactivations in preclinical MS, the increased gp350 seroreactivity demonstrated here may reflect the primary lytic infection or intermittent subclinical lytic reactivations of EBV^32^ perhaps representing an indirect indication of failing cellular immunity against EBV.^33^

Previous studies showed a lag of clinical MS after primary EBV infection. Serological studies from repositories showed EBV infection 1–2 decades before MS at the group level.^8 10^ Epidemiological studies showed an average lag of 10–20 years with wide distribution from IM to MS onset.^34 35^ We here show a decade of lag at the group level between the primary EBV infection and incipient neurodegeneration and a steady level of new primary infections during the preonset decade of increasing axonal injury. This indicates that MS pathology tends to evolve during the latency phase of an EBV infection. Pathology during the preclinical phase may result from secretion of inflammatory mediators (EBERS, several microRNAs), which occurs even in latency 0–1 of the EBV infectious cycle, when no EBV proteins are otherwise produced, or from non-proliferating reactivations. In subsequent MS pathology, such humoral factors may be relevant for cortical lesions without T-cell infiltrates and immunoglobulins at some distance from meningeal germinal centres.^36 37^ Furthermore, the lag between the primary EBV infection and MS-related axonal degeneration leaves time for possible influence by further exogenous risk factors for MS that also interact with EBV.^20 23 27 38^

According to contemporary opinion, MS is a central nervous system-specific autoimmune disease, although the search for an autoantigen has been unrewarding. Recently, cases with EBV-associated autoreactivity were identified in subsets of patients with MS. CSF serological analysis revealed cross-reactivity between a glial cell adhesion molecule and another EBNA1 epitope in 20%–25% of patients with MS.^39^ T-cell immunity depends on other EBV epitopes, and HLA-restricted CD4+T cells might be primed against two EBV-specific peptides and the CNS antigen RASGRP2.^40^ ANO2, a chloride channel protein that is important in several cell types and expressed in glial cells and neurons, has recently been investigated as an autoantigen in MS. ANO2 autoimmune reactivity in MS was demonstrated by screening a large number of antigens, and ANO2 antigen showed increased presence in the MS brain.^15^ Another previous study^16^ used a reciprocal absorption test showing that EBV antigen was able to inhibit antibodies induced by ANO2 and vice versa. Also, the cross-reactivity was confirmed with different assays, using both short peptides and a long ANO2 1–365 segments as antigen.^15 16^ The carriage of an ANO2 reactivity associated to an increased risk of MS in all types of combinations with HLA MS risk genes and EBNA1 reactivity,^16^ supporting a pathogenic role of this particular mimicry antigen. ANO2 is an intracellular antigen, and it is likely that it does not directly confer CNS damage, however, it may denote a pathogenic T cell reactivity. In this study, increased ANO2 seroreactivity did not appear until after EBV seroconversion and was limited to a subset of EBV-seropositive participants. Preonset ANO2 seroreactivity had an independent association with MS and with preonset axonal injury beyond the association with EBV infection. This relationship may result from a cross-reactive mechanism between EBNA1 and ANO2 as described in previous work.^15 16^ However, the ANO2 reactivity may also be an aspect of altered EBNA1 reactivity,^7^ with possible epitope spreading of the EBNA1 IgG antibodies. The importance of the altered reactivity, due to epitope spreading or cross-reactivity, is its association with increased preclinical sNfL, suggesting that it contributes to pre-MS axonal injury. Our finding of virtually no ANO2-seropositive individuals in EBNA1-seronegative groups in either the pre-MS or control cohorts may indicate the presence of a basal autoimmune diathesis in certain individuals in the general population.^16^ EBV-infected memory B cells may constitute a ‘forbidden clone’, with low but long-term propensity for autoimmunity.^41^

The strength of this study is the large repository material, matched with a national MS register, and sNfL assays performed in aliquots from the same specimens as latency and neutralising EBV activity and ANO2 reactivity assays. Our material captures a lengthy retrospective period extending well before clinical MS onset, incorporating the long average intervals between IM and MS of 10 years to decades.^34 35^

A weakness of this study is the lack of individual longitudinal data, which precluded observation of the exact individual sequence of seroconversions and sNfL. Data in the Swedish MS register are generally recorded by neurologists and quality controlled. However, some pre-MS samples were likely drawn during the MS prodrome with undetected subtle initial focal symptoms.^42^ This limitation is common to all studies of preclinical events. Still, this was not crucial for this study, which focused on changes in EBV seroreactivity presenting at least a decade before an objective neurochemical sign of MS.

A caveat is that the temporal relationships disclosed here do not exclude the presence of a confounder that could have been evoked by reduced function of natural killer, natural killer T or CD8+cells. Low natural killer cell numbers also have been associated with MS.^43 44^ Increased and atypical EBV serology in pre-MS may trace to a relatively more severe primary infection, either because of a higher viral load, possibly dependent on the oral infection pathway, or an insufficient CD8+T cell response.^33^ The subsequent control of the EBV survival also depends on cellular immunity, including CD8+lymphocytes activated as cytotoxic T lymphocytes. The HLA dependence of IM and MS may influence the pre-MS course through infection control in these phases.^45^

Because the level of sNfL decreases with higher body mass index (BMI)^46^ and adolescent overweight is a risk factor for MS,^47^ BMI is a source of possible bias. We did not have access to BMI data, but any such bias would have led to an underestimation of sNfL levels in this study.

The main results of this study are that EBV seroreactivity appeared a decade prior to the first signs of neuroaxonal injury, which in turn concentrated in the EBV seropositive group during the last decade before the clinical onset of MS. Furthermore, ANO2 antibodies, appearing in a subgroup shortly after primary EBV infection, were associated with preclinical neuroaxonal damage. These relationships implicate latent EBV infection in the pathogenesis of MS, at least as an interactive agent.

## Data Availability

Data are available on reasonable request. The data that support the findings of this study are available from the corresponding author, DJ, on reasonable request.
