# Supplementary material for: Seroreactivity against lytic, latent and possible cross-reactive EBV antigens appears on average 10 years before MS induced preclinical neuroaxonal damage
Source: J Neurol Neurosurg Psychiatry. 2023 Oct 6;95(4):325–32. doi: 10.1136/jnnp-2023-331868 (PMC10958269; doi:10.1136/jnnp-2023-331868)
Supplement: Supplementary data [file jnnp-2023-331868supp001.pdf]

Figure S1.

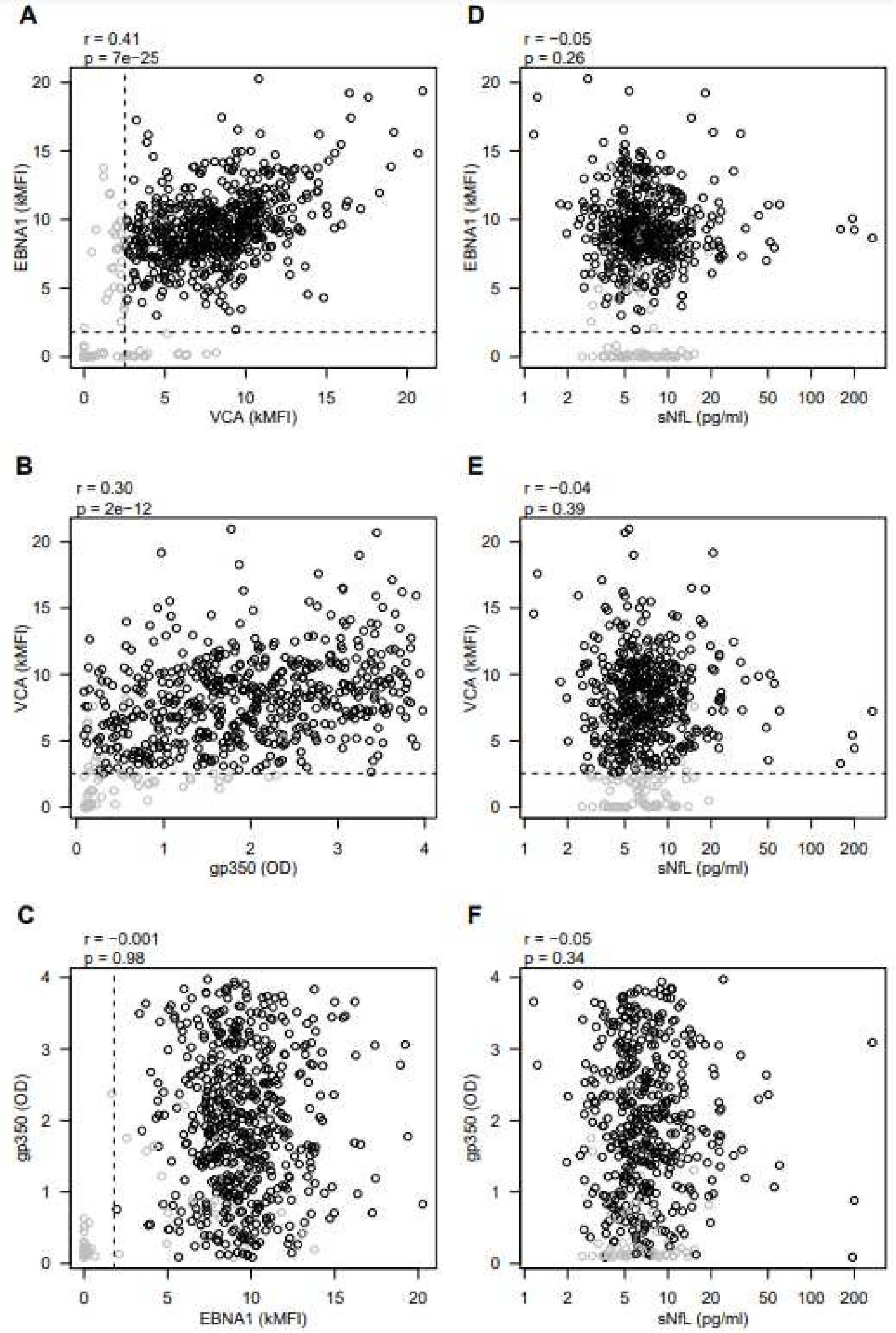

Figure S1. Intercorrelations between EBV antibodies and correlation with sNfL in pre-MS samples. Black circles indicate double (i.e., EBNA1 and VCAp18) seropositive pairs included in the correlation analyses in a–f; gray circles indicate pairs singly or not seropositive and not used for the present correlations; and dashed lines indicate cut-off for seropositivity as defined. (A and B) VCAp18 correlated weakly with EBNA1 and gp350. (C) No correlation was seen between EBNA1 and gp350 or between (D and F) sNfL and EBV serologies. Abbreviations: EBNA1, Epstein–Barr virus nuclear antigen 1; EBV, Epstein–Barr virus; gp350, glycoprotein 350; OD, optical density; pre-MS, before multiple sclerosis diagnosis; sNfL, serum neurofilament light; MFI, median fluorescent intensity; VCAp18, viral capsid antigen protein 18.

Figure S2.

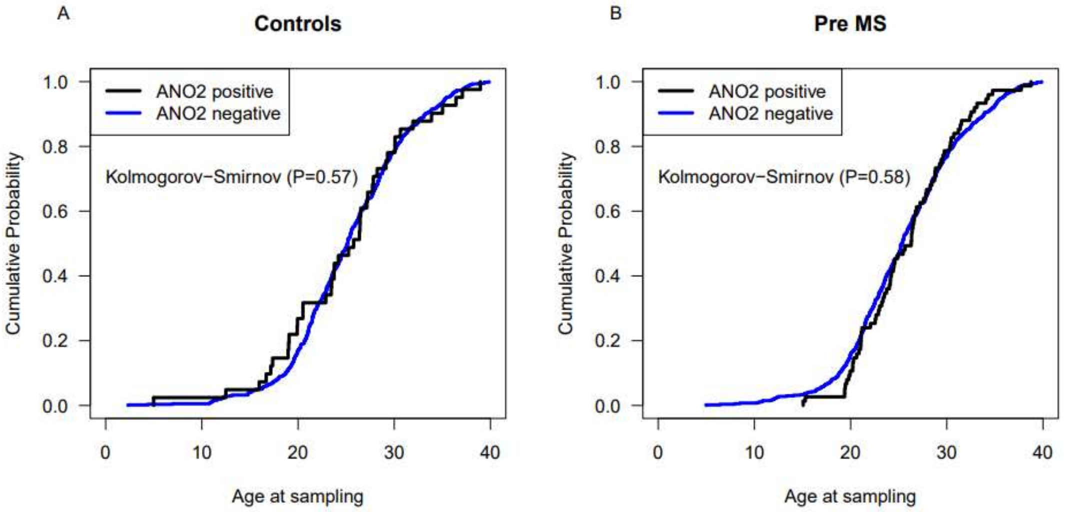

Figure S2. Age distribution of ANO2-seropositive and -negative samples within the EBV-seropositive group. (A) Controls. (B) Pre-MS cases. Abbreviations: ANO2, anoctamin 2; EBV, Epstein–Barr virus; pre-MS, before multiple sclerosis diagnosis.

Figure S3

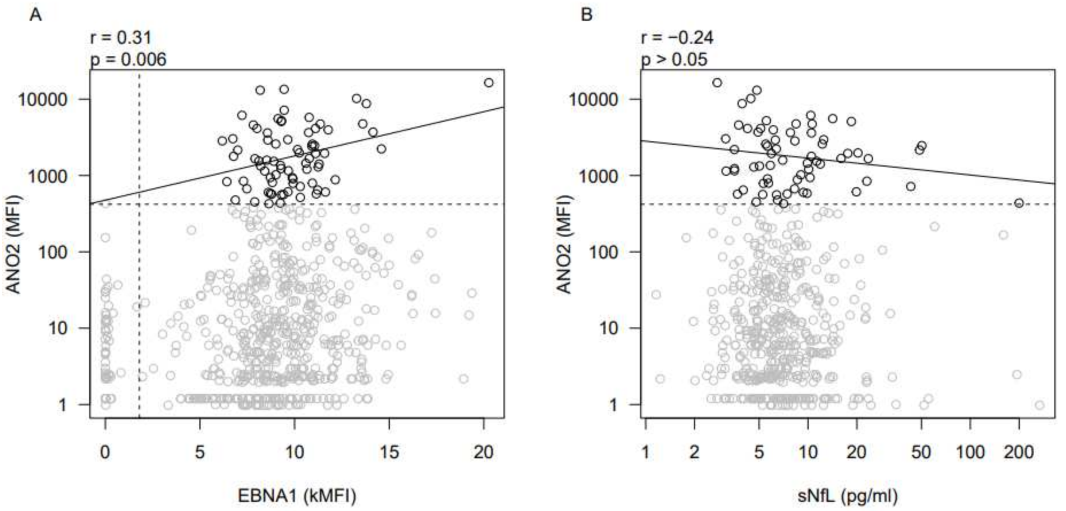

Figure S3. Correlation between ANO2 and EBNA1 antibodies and correlation with sNfL in pre-MS samples. Correlation coefficients were calculated for pre-MS samples seropositive for both ANO2 and EBNA1 (black circles). Correlation given in (A) included an outlier (far top right) and decreased to r=0.20 (p=0.09) after this data point was removed. Dotted lines indicate cut-off for ANO2 seropositivity as defined, and unbroken lines indicate regression on EBNA1 kMFI and sNfL with ANO2 kMFI as the dependent variable. ANO2, anoctamin 2; EBNA1, Epstein–Barr virus nuclear antigen 1; MFI, median fluorescent intensity; pre-MS, before multiple sclerosis diagnosis; sNfL, serum neurofilament light.
